# The Nucleocapsid Protein of Rift Valley Fever Virus Is a Potent Human CD8^+^ T Cell Antigen and Elicits Memory Responses

**DOI:** 10.1371/journal.pone.0059210

**Published:** 2013-03-18

**Authors:** Weidong Xu, Douglas M. Watts, Margaret C. Costanzo, Xiaolei Tang, Leon A. Venegas, Feng Jiao, Alessandro Sette, John Sidney, Andrew K. Sewell, Linda Wooldridge, Shinji Makino, John C. Morrill, Clarence J. Peters, June Kan-Mitchell

**Affiliations:** 1 Department of Biological Science and Border Biomedical Research Center, The University of Texas at El Paso, El Paso, Texas, United States of America; 2 Center for Infectious Disease, La Jolla Institute for Allergy and Immunology, La Jolla, California, United States of America; 3 Institute of Infection and Immunity, Cardiff University School of Medicine, Heath Park, Cardiff, United Kingdom; 4 Department of Microbiology and Immunology, The University of Texas Medical Branch, Galveston, Texas, United States of America; George Mason University, United States of America

## Abstract

There is no licensed human vaccine currently available for Rift Valley Fever Virus (RVFV), a Category A high priority pathogen and a serious zoonotic threat. While neutralizing antibodies targeting the viral glycoproteins are protective, they appear late in the course of infection, and may not be induced in time to prevent a natural or bioterrorism-induced outbreak. Here we examined the immunogenicity of RVFV nucleocapsid (N) protein as a CD8^+^ T cell antigen with the potential for inducing rapid protection after vaccination. HLA-A*0201 (A2)-restricted epitopic determinants were identified with N-specific CD8^+^ T cells from eight healthy donors that were primed with dendritic cells transduced to express N, and subsequently expanded *in vitro* by weekly re-stimulations with monocytes pulsed with 59 15mer overlapping peptides (OLPs) across N. Two immunodominant epitopes, VT9 (VLSEWLPVT, N_121–129_) and IL9 (ILDAHSLYL, N_165–173_), were defined. VT9- and IL9-specific CD8^+^ T cells identified by tetramer staining were cytotoxic and polyfunctional, characteristics deemed important for viral control *in vivo*. These peptides induced specific CD8^+^ T cell responses in A2-transgenic mice, and more importantly, potent N-specific CD8^+^ T cell reactivities, including VT9- and IL9-specific ones, were mounted by mice after a booster vaccination with the live attenuated RVF MP-12. Our data suggest that the RVFV N protein is a potent human T cell immunogen capable of eliciting broad, immunodominant CD8^+^ T cell responses that are potentially protective. Understanding the immune responses to the nucleocapsid is central to the design of an effective RVFV vaccine irrespective of whether this viral protein is effective as a stand-alone immunogen or only in combination with other RVFV antigens.

## Introduction

The RVFV, a *Phlebovirus* within the Bunyaviridae family, is a mosquito-borne zoonotic virus identified in 1930s in the Rift Valley of East Africa. It has a tripartite, negative single-stranded RNA genome. The L segment encodes a RNA-dependent RNA polymerase. The M segment encodes two glycoproteins (Gc and Gn) and two nonstructural proteins, the 78-kDa NSm1 and the 14-kDa NSm2. The S segment encodes a nonstructural NSs protein as well as the nucleocapsid protein (N). Both N and L proteins are required for viral replication and transcription. Gc and Gn proteins are incorporated into a viral envelope as glycoproteins, while ribonucleoprotein complex, which are formed by N and viral RNAs, and associated L proteins, are packaged into virions.

RVFV causes abortions and deaths in domestic ruminants, especially among young animals [1]. Transmission to humans occurs with bites from infected mosquitoes or through a break in the skin or aerosols during the handling of tissues of infected animals. Aerosol transmission was also reported for laboratory workers without appropriate protection [2]–[4]. Eighty percent of human infections display mild flu-like symptoms, and mortality rate was reported to be 0.5–1% due to diffuse hepatitis, hemorrhagic syndrome, and/or encephalitis [5]. However, higher fatality rates were reported in recent outbreaks, raising a concern that RVFV may pose a greater threat to public health than previously thought, especially in non-endemic regions [5].

RVFV has a genuine capacity to spread, with outbreaks in Egypt (1977), Western Africa (1988) and the Arabian peninsula (2000) [6]. It re-emerged after a long interval in Kenya (2006) and South Africa (2010). The presence of competent insect vectors, high viremia in infected animals, global changes in climate, and increased traffic to the African continent led to a consensus that RVFV outbreaks will eventually reach Europe and the United States [1]. The United States government also recognizes RVFV as a potential bioterrorism agent because of the high case-fatality rate and the potential for rapid spread [7].

There are no available commercially available vaccines for humans at this time [6], [8], although the formalin-inactivated RVFV TSI-GSD-200 is available under IND licensure for protection of military personnel and laboratory workers in the United States [9]. The live attenuated viruses, Clone 13 [10] and MP12 [11] are potential livestock vaccines [12]–[17], and MP12 was developed for use in humans but its safety profile remains to be completely validated. Inactivated vaccines, including one that successfully protected workers at high risk [9], [18], while safe are nonetheless expensive to produce and require multiple inoculations [9], [19].

Adaptive immunity induced by vaccinations with attenuated RVFV viruses, viral like particles (VLPs), or subunit vaccines can protect against lethal challenges in murine models [20]–[24]. The RVFV N protein elicits potent IgM and IgG responses that arise early after infection in humans and animals [25]–[28]. Of interest, N-subunit alone vaccines delivered as a recombinant protein [24], [29], [30] or a DNA vaccine [31]–[34] have been shown by independent laboratories to confer protection in the absence of detectable neutralizing antibodies (Abs) [29], [31], [34]. A role for N-specific T cells was implicated by the detection of dose-dependent proliferation of the spleen cells to N [31] and a rapid recall expression of Cd40, Cd40 ligand, Cd8a and Cd8b1 genes in the spleens of immunized mice, consistent with the activation of memory CD8 T cell immunity [30]. Finally, involvement of CD8^+^ T cells is consistent with the time course at which protection was acquired after a single VLP dose (10 d) [23], resembling the typical one-wave kinetics of virus-specific cytotoxic T lymphocytes (CTLs) after infection or vaccination [35].

As summarized above, there is compelling but indirect evidence that the RVFV N protein is a potent T cell immunogen that can protect against a lethal viral challenge in animal models, most likely through induction of virus-specific CD8^+^ T cell responses. Since T cells recognize viral epitopes in the context of the host MHC class I molecules, human T cells recognize a different spectrum of epitopes from their murine counterparts. Validation that the N protein is also immunogenic for humans is required. Because human testing is slow and costly, *in vitro* human modeling approaches can facilitate the development of new recombinant vaccines. We have developed *in vitro* immunization protocols to identify novel epitopes in the HIV proteome and to characterize the HIV-specific T cell responses [36]–[38]. Here this approach was used to map two shared, immunodominant CD8^+^ T cell epitopes encoded in N that are restricted by HLA-A2, one of the most common HLA alleles worldwide and to study the character of these epitope-specific CD8^+^ T cell repertoires.

## Materials and Methods

### Ethics Statement

A group of 8 HLA-A2^+^ healthy volunteers were enrolled after obtaining informed consent with approval from the Human Investigation Committee at The University of Texas at El Paso (Protocol 82702-7). HLA genotyping was performed by the Department of Transfusion Medicine, National Institutes of Health.

Eight- to ten-week-old C57BL/6-Tg (HLA-A2.1)1Enge/J (HLA-A2 transgenic) mice (Jackson Laboratory, ME) were bred in individually ventilated cages in a biosafety level 2 animal facility maintained by The University of Texas El Paso, Texas. All experiments were performed at biosafety level 3 according to experimental protocols (Protocol A-200911-1) approved by the Institutional Animal Care and Use Committee (IACUC) and the Institutional Biosafety Committee (IBC) and followed National Institutes of Health and United States Department of Agriculture guidelines.

### Lentiviral Vector and Transduction of Dendritic Cells (DCs)

A 0.7-kb cDNA fragment that encoded the whole N protein was amplified by the Polymerase chain reaction (PCR) using pT7-IRES-vN as a template and subcloned into the pLenti7.3/V5-TOPO plasmid (Invitrogen, CA). The sequence-verified recombinant vector, pLenti-vN was pseudotyped with vesicular stomatitis virus glycoprotein envelope (VSV-G). Viral stocks was produced according to manufacturer’s protocols (Invitrogen, CA). Virus titer was determined as the percentage of green fluorescent protein (GFP)-positive HT1080 cells 48 hours after transduction with serial dilutions of the lentiviral stock. Titers were expressed as transducing units (TU)/ml.

### Cells and Peptides

T2 [39] and C1R-A2^wt^ cells [40] expressing a full-length wild type human HLA-A2 were maintained in RPMI 1640 medium supplemented with 10% heat-inactivated fetal bovine serum. Peptides were purchased from Synthetic Biomolecules (San Antonio, TX). Fifty-eight 15-mer peptides overlapping by 11 residues (OLPs) across the entire N protein (GenBank Accession GU372973) were synthesized by Genemed Synthesis (San Antonio, TX). The carboxylic end (59^th^) OLP was only 13 residues in length. Lyophilized peptides were dissolved in DMSO (100 mg/ml) and stored as aliquots at −80°C.

### Generation of *in vitro*-primed N-specific CD8^+^ T cells from Healthy HLA-A2 Carriers

The procedure for generating CTL by *in vitro* immunization of naive circulating CD8^+^ T cells has been described [36]. Briefly, DCs were generated from adherence-purified peripheral blood monocytes by culturing for seven days in complete medium (RPMI 1640 with 10% autologous serum, 100 U/ml penicillin, 100 µg/ml streptomycin, 1 mM sodium pyruvate, 0.1 mM non-essential amino acids, and 2 mM L-glutamine) supplemented with GM-CSF (1000 U/ml, Leukine Sargramostim, Bayer HealthCare Pharmaceuticals, Montville, NJ) and IL-4 (500 U/ml, Peprotech, Rocky Hill, NJ). Cultures were replenished with GM-CSF and IL-4 every other day. Autologous CD8^+^ T cells purified from peripheral blood mononuclear cells by positive selection (Dynabeads; Invitrogen) were primed with irradiated (3000 cGy) DCs transduced at 5 TU/DC three days earlier by pLenti-vN at a T cell:DC ratio of 5∶1 in 48-well plates. CD8^+^ T cells were re-stimulated every seven to ten days thereafter with autologous monocytes pulsed with a pool of the 59 N OLPs [36], [37]. IL-7 (10 ng/ml, Genzyme, Cambridge, MA) was added on the day of priming and the day of each re-stimulation; IL-2 (20 U/ml, Peprotech) was added one and four days later.

To generate epitopic peptide-specific CD8^+^ T cells, purified CD8^+^ T cells were primed with autologous DCs pulsed for three hours with the cognate peptide (10 µg/ml) and re-stimulated every seven to ten days thereafter with fresh peptide-pulsed autologous monocytes [36].

### Mapping of Reactive OLPs by IFNγ ELISPOT Assays

OLP specificities in CD8^+^ T cell cultures were determined with the human IFNγ ELISPOT set according to the manufacturer’s instructions (BD Bioscience, San Jose, CA). Briefly, 50,000 CD8^+^ T cells were incubated for 16 hours with 200,000 C1R-A2^wt^ cells in the presence of 2 µg/ml of each peptide. Plates were developed with BD reagents to detect IFNγ production by peptide-specific T cells. The resulting number of spots was determined using the CTL ELISPOT Reader Unit (C.T.L., Shaker Heights, OH), and results were expressed as spot-forming cells (SFCs) per million input cells. The threshold for a positive response was considered to be at least 50 spots (1,000 SFCs/10^6^ CD8^+^ T cells) per well, and exceeding the mean plus three SDs of negative wells.

### Determination of the Binding Affinity of Epitopic Peptides to HLA-A2

Peptide binding affinity to HLA-A2 was determined by the inhibition of binding of a radiolabeled standard peptide [41]. Peptides were tested at six concentrations in three or more independent assays and the concentration that produced 50% inhibition (IC_50_) was calculated.

### Multiparametric Flow Cytometry to Character T cells

Directly-conjugated mAbs to CD8 (Qdot), CD107a (FITC-H4A3), CD107b (FITC-H4B4), IFNγ (PE-Cy7-4S.B3), IL-2 (PerCP-Cy5-MQ1-17H12), TNFα (Alexa Fluor 700-MAb11), and MIP-1β (PE-D21-1351) were purchased from BD Pharmingen (San Diego, CA). Intracellular cytokine production and degranulation were determined after a four-hour stimulation at 37°C with peptide-pulsed (10 µg/ml) T2 cells at a T cell:target cell ratio of 1∶1 [7]. Briefly, CD107a/b-FITC mAb and Golgi Stop/Golgi Plug (BD Biosciences) were added to the cells at the beginning of the incubation period. The cells were then washed with staining buffer (PBS containing 0.2% BSA and 0.02% sodium azide) and stained with the LIVE/DEAD Fixable Blue Dead Cell Stain (Invitrogen) and CD8-Qdot mAb for 30 min at 4°C. Next, the cells were permeabilized with Cytofix/Cytoperm (BD Biosciences) for 20 min at room temperature in the dark, washed twice with perm/wash buffer, re-suspended in staining buffer, and stored overnight at 4°C.

Intracellular staining was achieved by staining with antibodies for 30 min at 4°C. Cells were washed once in perm/wash buffer and re-suspended in fixed buffer. Staining was analyzed with a LSR II flow cytometer (BD Biosciences) and data analyzed by FlowJo Version 8.7.7 software (TreeStar, San Carlos, CA). Gating was performed on small lymphocytes, singlets, and viable CD8^+^ T cells. More than 10,000 CD8 events were collected for each sample.

### Peptide-specific T Cells by Staining with Tetrameric Peptide:HLA-A*0201 Complexes (Tetramers)

Monomeric IL9:HLA-A*0201 and VT9:HLA-A*0201 were provided by the National Institutes of Health/National Institute of Allergy and Infectious Diseases Tetramer Facility (Atlanta, GA). Tetramers were assembled by conjugating to fluorochrome-labeled streptavidin (BD Biosciences) according to the Tetramer Facility protocol. Cultured T cells were washed, re-suspended in cold staining buffer and stained with 1 µg/ml of tetramer and 100 ng/ml of Qdot-labeled anti-CD8 mAb for 30 min at 4°C. The cells were washed twice with staining buffer before analysis. Controls used to gate for specific tetramer binding included nonspecific staining of the cells under study by an irrelevant tetramer as well as exclusion of nonspecific binding of the tetramer under study to cultured T cells with irrelevant specificities.

### Tetramer Dissociation Assay

To compare avidity of binding of peptide-major histocompatibility complex class I (pMHCI) to T cell receptor (TCR), tetramer decay assays were conducted with N-specific CD8^+^ T cells in the presence of 10 µg/ml unconjugated anti-HLA-A2 antibody (clone BB7.2, Serotec) [42]. Briefly, peptide-specific CD8^+^ T cells were stained with an optimal concentration of cognate PE-conjugated HLA-A2 tetramer determined previously by titration in azide buffer (PBS containing 0.5% fetal bovine serum (FBS) and 0.1% sodium azide) for 20 min on ice to inhibit metabolically active shedding. Cells were then washed twice with ice cold azide buffer and dispensed into two aliquots and incubated at room temperature. To one sample, an excess of unconjugated BB7.2 antibody (100 µg/ml) was added to prevent tetramer from rebinding. Tetramer decay was then determined at time points 0, 1, 5, 20, 40, 60 and 90 min and analyzed by flow cytometry. The aliquot of cells incubated without BB7.2 antibody was used as a positive control and analyzed at 90 min.

### Cytotoxicity Assay

T2 target cells were labeled with sodium chromate (^51^Cr, PerkinElmer, Waltham, MA) and pulsed with an appropriate peptide for one hour at 37°C. After washing, the T2 cells were admixed with T cells at different E:T ratios in 96-well round-bottom plates. After an incubation period of four hours, supernatants were harvested and mixed with scintillation fluid (Optiphase SuperMix; PerkinElmer-Wallac, Gaithersburg, MD) and the amount of radioactivity determined with a MicroBeta counter (PerkinElmer-Wallac). T2 cells not pulsed with peptide or with an irrelevant peptide were used to control for spontaneous lysis. Specific percent lysis was calculated using the following formula: ((cpm experimental – cpm spontaneous)/(cpm total – cpm spontaneous))×100.

### Immunization of the HLA-A2 Transgenic Mice

C57BL/6-Tg (HLA-A2.1)1Enge/J (HLA-A2 transgenic) mice were immunized subcutaneously with 100 µg of epitopic peptide admixed with 100 µg of the pan-HLA-DR-binding peptide PADRE (aKXVAAWTLKAAaZC, X = L-cyclohexylalanine, Z = aminocaproic acid, [43]) emulsified in Incomplete Freund Adjuvant (IFA), given a booster vaccination ten days later by the same route and sacrificed on day 15. To detect peptide-specific precursors, splenocytes were harvested and re-stimulated *in vitro* at 1.5×10^6^ cells per ml with an equal number of irradiated syngeneic stimulator cells pulsed with cognate peptides (100 µg/ml for four hours) seven days in RPMI 1640 with 10% FBS and 55 nM of β2-mercaptoethanol. IL-2 at 40 I.U./ml was added on day 0 and day 4. Stimulator cells were syngeneic naive splenocytes pretreated for three days with 25 µg/ml lipopolysaccharide (LPS) (Sigma) at 2×10^6^ cells/ml. The LPS blasts were pulsed (25×10^6^ cells/100 µL) with 100 µM of the epitopic peptide for four hours in a 37°C and 5% CO2 incubator. Peptide-specific T cell responses were assessed by IFN-γ ELISPOT assay (BD Bioscience), chromium release assay, and flow cytometric determination of IFN-γ production.

To determine whether N-specific CD8^+^ T cells were induced by natural RVFV infection, HLA-A2 transgenic mice (N = 3) were immunized subcutaneously with 1x10^4^ PFUs of the live attenuated MP-12 virus [44]. Mice received a booster vaccination at the same dose six weeks later. Five days thereafter, splenocytes were isolated and re-stimulated for six hours with C1R-A2-wt cells pulsed with N OLP pool (10 µg/ml for each OLP), VT9 (10 µg/ml) or IL9 peptide (10 µg/ml). N-specific CD8^+^ T cells secreting IFN-γ were analyzed by intracellular cytokine staining. Cultured splenocytes stimulated by phorbol 12-myrstate 13-acetate (PMA, 10 ng/ml, Sigma, St. Louis, MI) and ionomycin (1 µM, Sigma) and unstimulated splenocytes were included as positive and negative controls, respectively.

## Results

### Expression of N Protein by the pLenti-vN-transduced DCs

**Figure 1:**
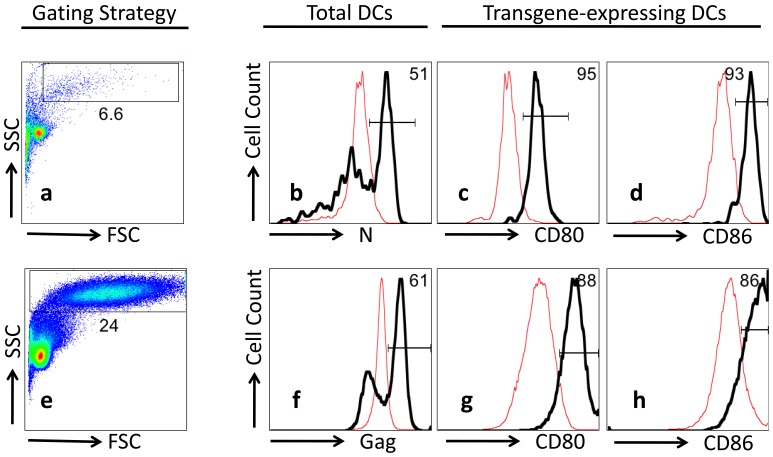
Expression of N protein by pLenti-vN-transduced DCs. DCs were cultured from monocytes in complete medium supplemented with GM-CSF and IL-4 for four days and transduced with a lentiviral vector encoding RVFV N (**Panels a**, **b**, **c** and **d)** or HIV Gag (**Panels e**, **f**, **g** and **h**). DCs not exposed to the vector were set aside as negative (untreated) controls. Expression of transgene was assessed on day 7 by intracellular staining with N-specific mAb S-370 or Gag-specific mAb KC57. **Panels a** and **e** show the gating of DCs by forward and side scatter. **Panels b** and **f** show 51% and 61% of the DCs to expression N and Gag, respectively. Nearly all DCs expressing transgene products underwent further maturation based on increase expression of CD80 (**Panels c** and **g**) and CD86 (**Panels d** and **h**).

We have previously reported that human monocyte-derived DCs transduced by multiply deleted HIV-1 vectors expressing various transgenes retained their typical phenotypes and functions, including the ability to prime antigen-specific cytotoxic T cell response *in vitro* [45]. Here we constructed a lentiviral vector, pLenti-vN to express RVFV nucleocapsid N. Intracellular N protein expression was verified by staining transduced DCs with the mouse anti-N mAb S-370. **Figure 1a** shows that DCs were easily distinguished from small lymphocytes by FSC and SSC as large granular cells in DC cultures. **Figure 1b** shows overlapping histograms of transduced and control (“non-transduced”) DCs from a donor (heavy and light lines, respectively). Fifty one percent of DCs within the transduced culture stained positively for N-expression. The remaining cells did not express N, as indicated by DCs in the corresponding untreated culture. **Figure 1c and 1d** indicate that N-expressing DCs were more mature, with higher expression of the co-stimulator molecules, CD80 and CD86. **Figure 1e, 1f, 1g and 1h** summarize a parallel experiment showing that DCs transduced to express HIV Gag protein were also matured immunologically. Incidentally, there was significant day-to-day and donor-to-donor variation in the percentages of transduced DCs, which can range from 28 to 62%.

### Immunodominant Epitopes of the N Protein Identified by *in vitro* Immunized CD8^+^ T cells

CD8^+^ T cells purified from eight HLA-A2^+^ healthy donors were primed with autologous DCs expressing the N protein and re-stimulated weekly thereafter with autologous monocytes pulsed with 59 OLPs spanning this protein. Reactivities to individual OLPs were assessed on day 30–42 by IFNγ-ELISPOT assays. OLPs 41 and 42, OLPs 30 and 31, and OLP 20 were judged likely to encode immunodominant epitopes based on their almost universal recognition by the cell cultures and the high frequencies of their IFNγ responses (**Table 1**). Another 19 OLPs were also recognized (**Table 1**). Since these reactivities were lower in general and the responses observed in only one to four cultures, these OLPs were mostly likely subdominant. It should be noted that “immunodominance” of OLPs may be determined by how efficiently particular 15-mers are spontaneously trimmed to optimal epitopic peptide lengths by serum peptidases *in vitro*. Regardless, these data show the existence of robust human CD8^+^ T cell repertoires specific for both public and private epitopes encoded by the RVFV N protein. Moreover, since transduced DCs consistently primed robust N-specific T cell responses *in vitro*, this RVFV protein is likely highly immunogenic to humans.

**Table 1:** Classification of immunodominant and subdominant reactivity to OLPs in eight N-specific CD8^+^ T cell cultures from different HLA-A*0201^+^ donors.

Donor I.D.	Reactivity to an individual OLP																							
	41	42	30	31	20	57	1	6	9	24	28	45	54	2	5	8	19	22	25	28	43	47	48	58
UT-24	3+	3+	3+	3+	2+	2+				1+	2+	1+	1+								1+			
UT-1	3+	3+	2+	2+	2+	2+	1+		1+					1+		1+								
UT-25	2+	2+	2+	2+	1+			1+																
UT-26	2+	2+	2+	1+																				
UT-5	1+	2+	2+	2+		2+	1+				1+											1+	1+	
UT-11	3+	3+			3+				1+	2+		1+	2+		1+				1+	1+				
UT-7	1+	2+			2+			2+									2+	1+						
UT-20			2+	2+	1+	2+																		2+
# Reactive/Tested	7/8		6/8			4/8	2/8							1/8										
	Immunodominant					Subdominant																		

3+, 2+ and 1+ denote >600,100 to 600, and 50–100 SFCs/50,000 T cells in each well, respectively.

Minimal epitopes within reactive OLPs were mapped by the ELISPOT assay with a panel of shorter peptides that were progressively truncated by one residue from either the C- or the N-terminus (**Table 2**). With this approach, an optimal 9-mer epitope VT9 (VLSEWLPVT, residues 121–129) was defined in OLP20. Of interest, OLP42 appeared to encode two overlapping 9-mer epitopes: IL9 (ILDAHSLYL, residues 165–173), and LL9 (LDAHSLYLL, residues 166–174).

**Table 2:** Fine mapping of minimal epitopes in OLP31 and OLP42.

Culture	Peptide #	Sequence															SFCs/50,000 T cells	
UT-20	OLP31	V	L	S	E	W	L	P	V	T	G	T	T	M	D	G	229	
	31-1	V	L	S	E	W	L	P	V	T	G	T	T	M	D		141	
	31-2	V	L	S	E	W	L	P	V	T	G	T	T	M			157	
	31-3	V	L	S	E	W	L	P	V	T	G	T	T				138	
	31-4	V	L	S	E	W	L	P	V	T	G	T					125	
	31-5	V	L	S	E	W	L	P	V	T	G						108	
	31-6	**V**	**L**	**S**	**E**	**W**	**L**	**P**	**V**	**T**							116	
	31-7	V	L	S	E	W	L	P	V								8	
	31-8		L	S	E	W	L	P	V	T	G	T	T	M	D	G	4	
	31-9			S	E	W	L	P	V	T	G	T	T	M	D	G	0	
	31-10				E	W	L	P	V	T	G	T	T	M	D	G	2	
	31-11					W	L	P	V	T	G	T	T	M	D	G	4	
	31-12						L	P	V	T	G	T	T	M	D	G	4	
	31-13							P	V	T	G	T	T	M	D	G	2	
UT-11	OLP42	I	L	D	A	H	S	L	Y	L	L	Q	F	S	R	V	>600	
	42-1	I	L	D	A	H	S	L	Y	L	L	Q	F	S	R		>600	
	42-2	I	L	D	A	H	S	L	Y	L	L	Q	F	S			>600	
	42-3	I	L	D	A	H	S	L	Y	L	L	Q	F				>600	
	42-4	I	L	D	A	H	S	L	Y	L	L	Q					>600	
	42-5	I	L	D	A	H	S	L	Y	L	L						>600	
	42-6	**I**	**L**	**D**	**A**	**H**	**S**	**L**	**Y**	**L**							>600	
	42-7		L	D	A	H	S	L	Y	L	L	Q	F	S	R	V	>600	
	42-8			D	A	H	S	L	Y	L	L	Q	F	S	R	V	11	
	42-9				A	H	S	L	Y	L	L	Q	F	S	R	V	23	
	42-10					H	S	L	Y	L	L	Q	F	S	R	V	43	
	42-11						S	L	Y	L	L	Q	F	S	R	V	18	
	42-12							L	Y	L	L	Q	F	S	R	V	1	
	OLP42	I	L	D	A	H	S	L	Y	L	L	Q	F	S	R	V	>600	
	42-7.1		L	D	A	H	S	L	Y	L	L	Q	F				>600	
	42-7.2		L	D	A	H	S	L	Y	L	L	Q					>600	
	42-7.3		**L**	**D**	**A**	**H**	**S**	**L**	**Y**	**L**	**L**						>600	
	42-7.4		L	D	A	H	S	L	Y	L							120	

The above data showed the number of SFCs/50,000 T cells for a representative CD8^+^ T cell culture after stimulation by the corresponding truncated peptide. Each culture was tested at least twice on different days and the optimal minimal epitopes were deconvoluted based on three different cultures with cognate reactivity.

Consistent with their immunogenicity, VT9 and IL9 bind to purified HLA-A2 with good affinities, with IC_50_ values of 15 nM and 1.3 nM, respectively (**Table 3**). Thus, both peptides bind to HLA-A2 as strongly as the reference FV10 epitope [46], and with an affinity that is greater by one log as compared to that of the immunodominant HIV Gag epitope, SL9 [47].

**Table 3:** Binding affinity of VT9 and IL9 to HLA-A*0201.

Peptide	Amino Acid Sequence	IC50 (nM)
VT9	VLSEWLPVT	15.0
IL9	ILDAHSLYL	1.3
SL9^(1)^	SLYNTVATL	79.0
FV10^(2)^	FLPSDYFPSV	5.0

1 SL9 is the well characterized epitope in HIV Gag p17 (position 77–85).

2 FV10 is an epitope of the HBV core antigen (position 18–27).

### Immunological Characteristics of VT9- and IL9-specific CD8^+^ T cells

**Figure 2:**
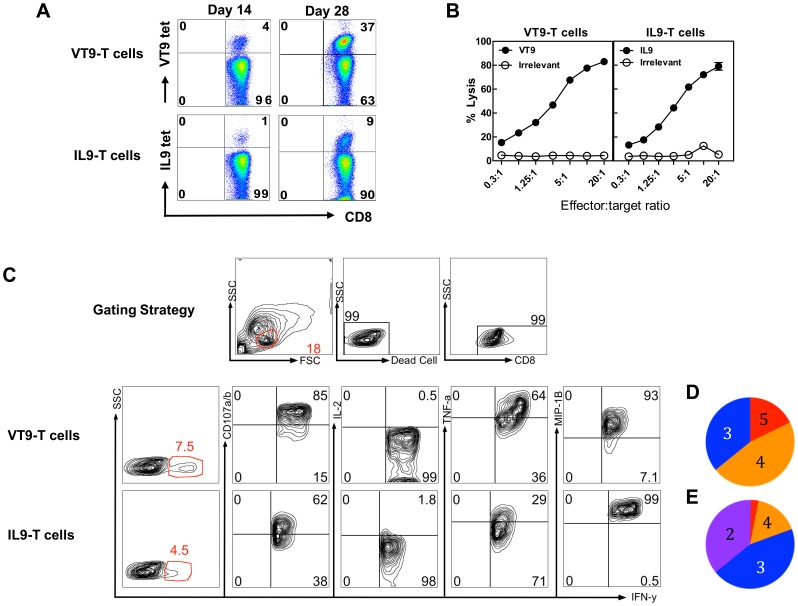
Characteristics of VT9- and IL9-specific CD8^+^ T cells. **Panel A** shows progressive increase of VT9- and IL9-specific CD8^+^ T cells with culture. **Panel B** shows these epitope-specific CD8^+^ T cells to be cytotoxic as judged by the chromium release assay over a range of E:T ratios (0.3 to 20∶1) using T2 target cells pulsed with 10 µg/ml peptides. **Panel C** shows a representative gating strategy used to isolate viable CD8^+^ small lymphocytes in the cultures and the dot plots of IL9- and VT9-specific T cells defining the IFNγ^+^ subset were polyfunctional as shown by polychromatic flow cytometry (degranulation by surface expression of CD107a/b) and secretion of IL-2, TNFα, and MIP-1β). **Panel D** and **E** are pie charts summarizing the four possible functional outcomes according to the number of additional functions for IFNγ-producing VT9- and IL9-specific T cells, respectively. Pie slices represent the proportion of responding CD8^+^ T cells that upregulated 5 (red), 4 (orange), 3 (blue), and 2 (purple) function(s).

To study the immunological characteristics of VT9- and IL9-specific CD8^+^ T cells, CD8^+^ T cells specific to VT9 or IL9 were generated from a healthy HLA-A2 carrier. Purified circulating CD8^+^ T cells were primed for seven days with either VT9- or IL9-pulsed DCs and re-stimulated weekly thereafter with autologous monocytes pulsed with the cognate peptide. Expansion of peptide-specific T cells was assessed by tetramer staining. **Figure 2A** shows increasing percentages of tetramer-binding T cells over time (days 14 and 28). VT9-specific T cells increased from 4 to 37% over this period, while IL9-specific T cells increased from one to 9%. Of note, tetramer-binding CD8^+^ T cells in both cultures appeared as a well-delineated and seemingly homogeneous population that was easily resolved from non-specific counterparts.

Qualitative attributes of virus-specific CD8^+^ T cells appear to correlate with the efficacy of immune control of infection [48], [49]. The ability of CD8^+^ T cells to specifically recognize and rapidly destroy infected cells is important to limit further dissemination. As shown in **Figure 2B**, VT9- or IL9-specific CD8^+^ T cells are highly cytotoxic, specifically lysing C1R-A2^wt^ target cells pulsed with the cognate but not an irrelevant peptide over a range of E:T ratios.

Polyfunctionality, the ability of CD8^+^ T cells to simultaneously display a number of effector functions (such as degranulation and production of immune or antiviral factors) upon encountering antigen [50] is viewed as an important correlate of T cell-mediated immune control in infectious diseases [51]. Here, polyfunctionality of VT9- and IL9-specific CD8^+^ T cells was analyzed by multiparametric flow cytometry to ascertain degranulation (surface expression of CD107a/b [52]) and intracellular production of IFNγ, TNFα, IL-2 and MIP-1β after specific stimulation. As shown in **Figure 2C**, various proportions of VT9- and IL9-specific CD8^+^ T cells identified by production of IFNγ after antigenic stimulation were CD107a/b^+^, IL-2^+^, TNFα^+^ and MIP-1β^+^. **Figures 2D and 2E** summarize the functional response profiles of T cells based on the number of functions in pie charts. Peptide-specific T cells were polyfunctional, although VT9-specific T cells as a whole showed higher functionality than IL9-specific cells.

### Binding Affinity of VT9- or IL9-specific TCRs to Cognate peptide:MHCI (pMHCI) Complexes

**Figure 3:**
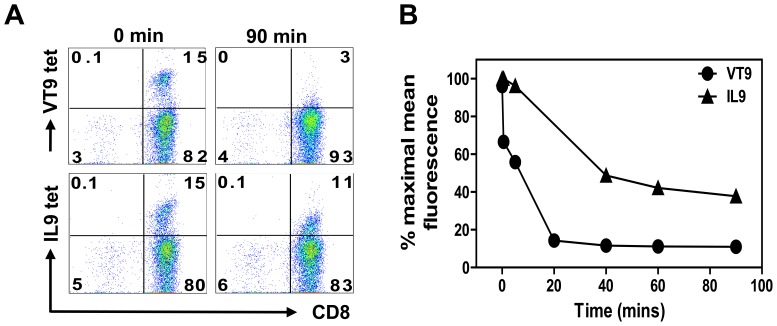
Differences in TCR/pMHCI dissociation. **Panel A** shows the reduction of VT9: and IL9:tetramer binding over 90 min in the presence of excess anti-HLA-A2-specific mAb BB7.2 to prevent reattachment of cognate peptide-specific CD8^+^ T cells. **Panel B** shows decreasing percent maximal mean fluorescent staining during this period.

The rate of dissociation of pMHCI tetramers from the cell surface of CD8^+^ T cells correlates with their binding affinities to the TCRs [42]. **Figure 3A and 3B** show the differential shedding of bound cognate tetramers on VT9- and IL9-specific T cells (from 15 to 3% and 15 to 11%, respectively), and the progressive reduction in mean fluorescent staining over 90 min at room temperature. The observation that tetramers to two immunodominant N protein determinants can bind with substantially different affinities indicates that factors in addition to the triggering of TCR by pMHCI ligands contribute to their immunodominance.

### VT9 and IL9 as Peptide Vaccines in the HLA-A2 Transgenic Mice

**Figure 4:**
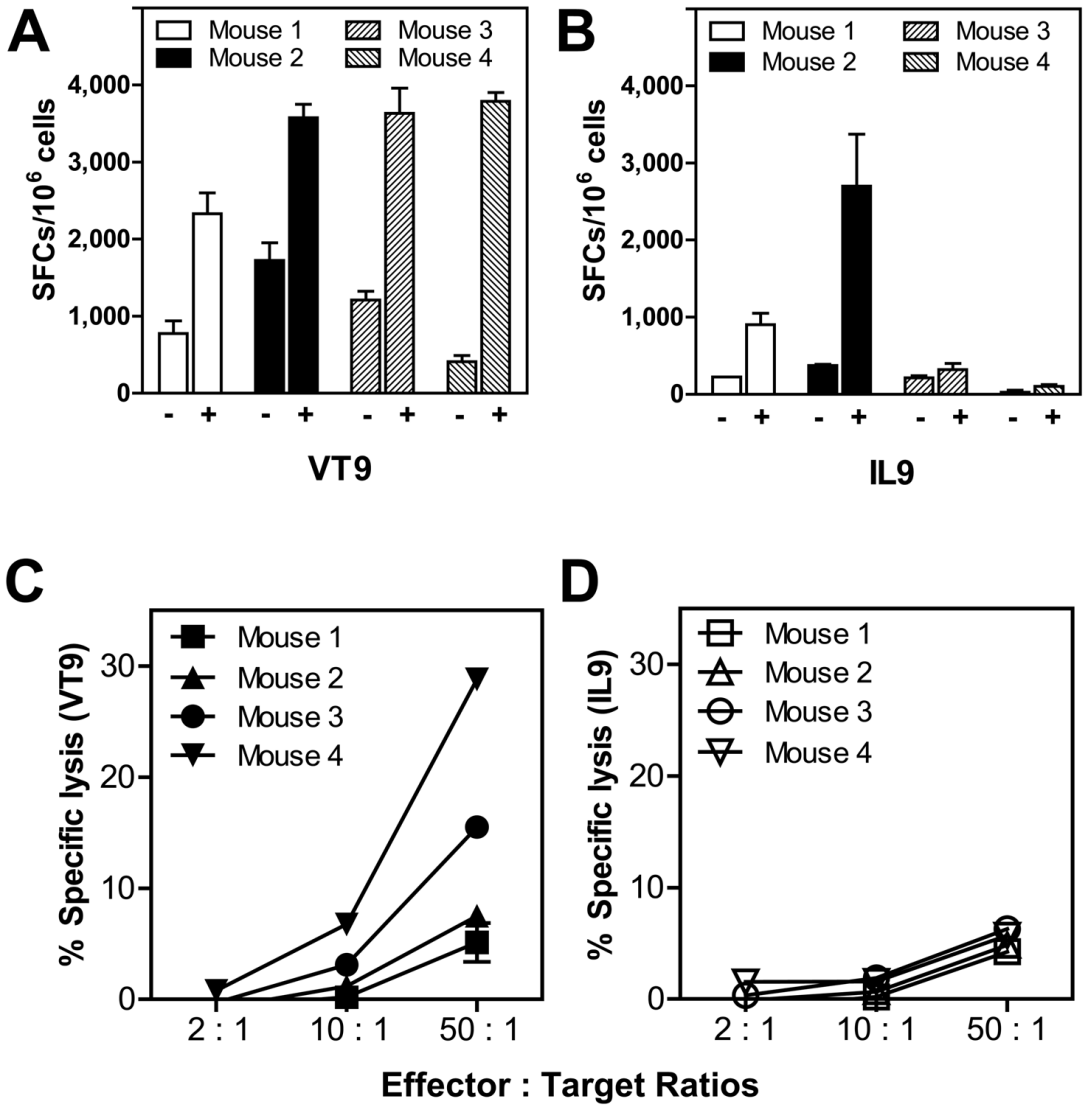
Immunization of HLA-A2 transgenic mice with epitopic N peptides. Two groups of four mice each were immunized subcutaneously and boosted ten days later with 100 µg of VT9 or IL9 emulsified with 100 µg PADRE in IFA. Splenocytes were collected on day 15 and re-stimulated *in vitro* with cognate peptide-pulsed irradiated syngeneic LPS blast cells for seven days. Peptide-specific responses were assessed by IFNγ ELISPOT and chromium release cytotoxicity assays. **Panels A and B** show the number of SFCs per million splenocytes from mice immunized with VT9 or IL9, respectively with and without the cognate peptide. **Panels C and D** show the percent specific lysis of cognate peptide-pulsed T2 target cells mediated by cultured splenocytes.

The ability of N peptides to stimulate *de novo* CD8^+^ T cell precursors in HLA-A2 transgenic mice without prior virus exposure was evaluated. Mice were primed and boosted once with VT9 or IL9 admixed with the PADRE pan-T helper cell epitope and emulsified in IFA. Splenocytes were re-stimulated by culturing with syngeneic B cell blasts pulsed with cognate peptides for 7 days. The frequencies of specific IFNγ-secreting cells were determined by the ELISPOT assay (**Figure 4A and 4B**). VT9-specific responses were found in four of four mice immunized. Significant IL9-specific responses were noted only in two of four mice and were generally lower in magnitude than that observed for VT9, reminiscent of their relative representation in human *in vitro* peptide-primed CD8^+^ T cultures (**Figure 2A**). This finding suggests unequal precursor T cell pool sizes for these immunodominant epitopes in humans and mice.

To characterize these peptide-specific responses further, splenocytes re-stimulated one cycle *in vitro* were examined for their ability to specifically lyse cognate peptide-pulsed T2 target cells at various E:T ratios. As shown in **Figure 4C**, VT9-specific killing was mediated by T cells from mice immunized with this peptide. Moreover, the level of cytotoxicity was comparable to that elicited by a potent cytomegalovirus peptide vaccine in a similar humanized model [53]. In contrast, no peptide-specific cytotoxicity was detected in re-stimulated splenic T cells from all four mice immunized with IL9 (**Figure 4D**). Discordant cytokine secretion and cytolysis upon encountering the cognate antigen is not uncommon among effector CD8^+^ T cells [54]. Interestingly, there was no association between the magnitude of the CD8^+^ T cell responses and the HLA-A2 binding affinities of the epitopes (IC_50_ of 1.3 nM for IL9 and 15.0 nM for VT9) [55], [56]. This may be explained by differing affinities for TAP transporters [57] and for Tapasin [58], relative frequencies of naïve T cell precursors [59], and perhaps even selective culling of IL9-specific responses [36], [60].

### Robust N-specific CD8^+^ T cell Responses after Vaccination with an Attenuated RVFV (MP-12 Strain)

**Figure 5:**
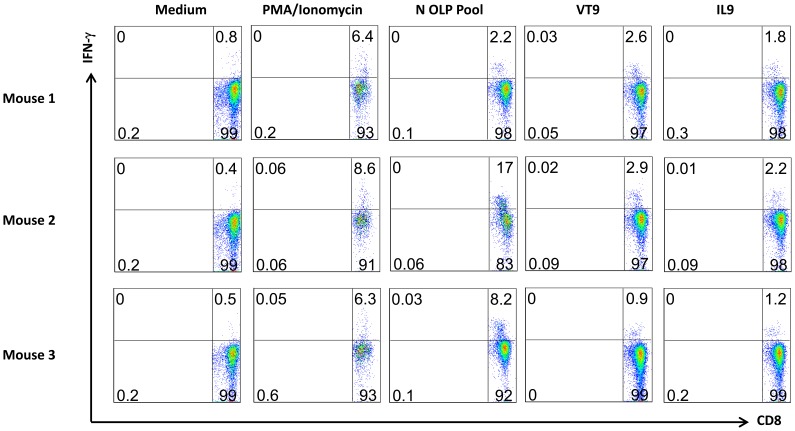
Robust N-specific CD8 T cells induced by the protective attenuated RVFV MP-12 vaccine in HLA-A2 transgenic mice. Mice were immunized subcutaneously with 1x10^4^ pfu of MP-12 and boosted with the same dose six weeks later. Splenocytes were harvested five days later and re-stimulated with T2 cells pulsed with N-OLP peptide pool (10 µg/ml each), VT9 or IL9 peptide (10 µg/ml) for 6 hours. Splenocytes treated with PMA (10 ng/ml) and Ionomycin (1 µM) (PMA/Ion) or cultured in medium without peptide were used as positive and negative controls, respectively. N-specific CD8^+^ T cell responses were assessed by the secretion of intracellular IFNγ.

MP-12 is an attenuated virus derived from RVFV ZH548 carrying nine point mutations across all three RNA segments but none within the N gene [8]. Here, robust N-specific CD8^+^ T cell responses (2 to 16% of total CD8^+^ T cells) were detected in splenocytes of mice immunized twice with MP-12 as determined by intracellular IFNγ staining (**Figure 5**). Remarkably, potent CD8^+^ responses to the VT9 and IL9 epitopic peptides were also detected, consistent with their immunodominant stature. Moreover, they suggest that the epitopes are naturally processed and presented by MP-12-infected antigen presenting cells. The potential of VT9 and IL9 being included for vaccine design will also depend on their natural processing and presentation following RVFV infection.

## Discussion

There is compelling evidence from several laboratories that stand-alone RVFV N-subunit vaccines can protect against lethal wild type viral challenges in the mouse [24], [29], [31], [61]. Since the internal N protein would not induce antibodies capable of neutralizing viral particles, we postulate that the protection was mediated by T cells. Indeed, structural proteins of most viruses appear to consistently elicit potent host anti-viral CD8^+^ T cell responses that can limit infections. Since these are usually well-conserved proteins expressed at high levels in early infections, they are often considered important targets for T cell-based vaccines [62]. Here we described two HLA-A2-restricted immunodominant epitopes (VT9 and IL9) across the RVFV N protein identified by *in vitro* immunized N-specific CD8^+^ T cells from healthy donors. Because the T cells were primed by autologous DCs transduced to express the N protein, we surmised that the epitopes are naturally processed and presented. Both epitopic peptides displayed high binding affinities to the HLA-A2 class I molecule by a quantitative binding assay, a property characteristic of many potent CD8^+^ T cell epitopes. CD8^+^ T cell lines generated by *in vitro* discontinuous cycles of stimulation with cognate peptide-pulsed APCs were polyfunctional, a quality of T cells heralded as a correlate of protection. Of most physiological relevance is the detection of a robust N-specific recall CD8^+^ T cell response after mice received a booster vaccination of the protective live MP-12 RVFV vaccine strain, showing for the first time that the N protein is a key T cell target during RVFV infection. Of note, both VT9 and IL9 reactivities were prominently represented indicating that these epitopes are naturally processed and presented. In sum, our data are consistent with the well-established findings for influenza A virus, where CTLs targeting the conserved internal viral nucleoprotein have been shown to contribute to protective heterosubtypic immunity (reviewed in [63], [64]). Moreover, systemic vaccination with this protein accelerates viral clearance and prevents death after challenges with various influenza serotypes [65], [66].

Our data demonstrated that N protein is a key T cell immunogen during RVFV infection. Although antiviral CD8^+^ T cells do not prevent infections, they promote virus clearance via production of pro-inflammatory cytokines and direct killing of virus-infected cells, thereby limiting dissemination and reducing host morbidity [67]. Memory T cells generated from vaccination are also capable of rapid recall to effector functions to prevent re-infection. The primary immunological correlate for effective vaccines to many human viral pathogens is neutralizing antibodies, which effectively bind to envelope molecules presented on free virions and infected cells. It should be noted that non-neutralizing antibodies can also be protective, albeit to a lesser degree and directed primarily at infected cells [68]. It is becoming apparent that immune correlates of protection for complex viruses with complicated lifecycles or with high mutation rates including, perhaps RVFV may be multifactorial, involving a combination of innate and adaptive T and B cell immunity [69]. While antibodies to the envelope proteins of influenza virus are the primary correlates of protection, pre-existing CD8^+^ T cell responses can provide a degree of protection against newly emerging viruses because they recognize the more conserved internal components, thereby blunting the severity of infections by serologically distinct strains, for which minimal antibody immunity exists [64], [70]. Here we showed that the highly conserved RVFV N protein [71] induces potent CD8^+^ T cell responses. The cumulative impact afforded by even a small increase in temporary control and the attendant reduction of secondary transmission may be substantial to limiting the spread of a severe epidemic.

As with other negative-sense RNA viruses, the RVFV N protein binds with some affinity to the viral genomic RNA (encapsidation), thereby providing a protective protein coat. It also plays an essential role in several steps within the viral replication cycle [72]. It is the most abundant viral component of the virion [28] and has been shown to be highly conserved among RVFV isolates sequenced so far [71]. Because high serum titers of N-specific IgM antibodies are engendered early post infection in both human and animal hosts, N has been a favorite antigen choice for diagnostic assays [25], [26], [73], [74]. Whether N-immune IgM or IgG antibodies contribute to the control of RVFV infection has received little or no attention, despite numerous examples of protection against enveloped viruses after passive transfer of non-neutralizing antibodies [68]. Recent data show that adoptively transferred IgG antibodies to the influenza nucleoprotein protect naïve mice [75], [76]. Intriguingly, virus clearance depends on the FcRs of the antibodies and cooperation with CD8^+^ cells [77]. Studies in autoimmune diseases [78] showed that “interferogenic” immune complexes of IgG and self-nucleic acids internalized via FcγRIIA (CD32A) to endosomes by plasmacytoid DCs (pDCs) where they engage Toll Like receptors (TLRs) to activate production of inflammatory cytokines and type I interferons [79]. Immune complexes formed with influenza nucleoprotein or RVFV nucleocapsid in tandem with cognate viral genomic RNA are likely to activate pDCs in a similar fashion, perhaps through TLR7. IFNα is directly antiviral and is a potent immunological adjuvant that promotes maturation of DCs, polarization of Th cells to Th1 cells, and activation of CTLs [79]. Of note, not all internal viral proteins are good non-neutralizing antibody targets, since vaccination with the influenza nonstructural 1 protein was not protective [77]. It is possible that intrinsic immunogenicity to CD8^+^ T cells is also crucial.

In terms of vaccine design, the value of N and other internal viral proteins as vaccine targets is only now becoming appreciated. It is well-established based on work from independent laboratories [24], [29]–[34] that an N protein vaccine can confer protection, although the protection afforded has generally been assumed to be less effective than that after vaccination with glycoprotein vaccines. The inclusion of a T cell-component to a RVFV vaccine has other theoretical advantages. CD8^+^ T cells directed at conserved N epitopes would complement the efficacy of antibodies targeting the surface glycoproteins, particularly Gc [6], [80], which is a highly variant protein with 2.2% amino acid substitutions [81]. RNA viruses are mutable due to a low fidelity RNA polymerase and therefore, differences in antigenicity can potentially exist among different RVFV lineages and quasispecies. While it remains to be determined whether host immunological pressures contributed to the genetic diversity of RVFV [82], there is precedence in other viral infections that a single amino acid residue change within a key viral epitope is sufficient to severely impair immune recognition by T or B cells. In HIV-1 infections, there are numerous examples of highly specific T cell receptors sensitive to single amino acid changes [83] as well as compelling evidence of HIV-1 variants escaping existing T cell responses in infected individuals by single mutations in epitopes [84]. Indeed, the HIV-1 quasispecies within a single infected person arise from a single transmitted founder virus through rapid accumulation of mutations in regions encoding CD8 T cell epitopes [85]. Neutralizing antibodies induced by immunization against hepatitis B infection are targeted to immunodominant conformational epitopes in the ***α*** determinant, which spans amino acids 124–147 of the surface antigen. However, viral variants encoding amino acid substitutions within this region of the surface protein, including a single substitution from glycine to arginine at amino acid position 145 (G145R) are not recognized by vaccine-induced antibodies, effectively abrogating vaccine efficacy [42]. Therefore, harnessing different aspects of anti-viral adaptive immunity to offer protection against a range of circulating viral strains, and potentially an emergent pandemic strain appears to be a prudent approach.

RVFV vaccines should trigger cross-protective T cell immunity against a broad spectrum of RVFV isolates while inducing effective neutralizing antibodies. Here we have shown that the nucleocapsid which provokes potent serum antibody responses after *in vivo* infections is highly immunogenic to CD8^+^ T cells as well. From the perspective of T cell immunity, future studies should examine its immunogenicity to CD4^+^ T cells, since CD4^+^ T cells to influenza core proteins correlated with disease limitations [86]. Further studies are needed to determine whether future vaccines for RVFV that generate immunity to the nucleocapsid protein may prevent or limit infection by this complex RNA virus.
